# Lean-Proteome Strains – Next Step in Metabolic Engineering

**DOI:** 10.3389/fbioe.2015.00011

**Published:** 2015-02-06

**Authors:** Kaspar Valgepea, Karl Peebo, Kaarel Adamberg, Raivo Vilu

**Affiliations:** ^1^Competence Center of Food and Fermentation Technologies, Tallinn, Estonia; ^2^Department of Chemistry, Tallinn University of Technology, Tallinn, Estonia; ^3^Department of Food Processing, Tallinn University of Technology, Tallinn, Estonia

**Keywords:** strain engineering, recombinant cells, absolute quantitative proteomics, genome engineering, *Escherichia coli*, genome reduction, chassis cells, whole-cell model

Rapid development of high-throughput -omics (e.g., proteomics) and genetic engineering technologies together with an array of new metabolic modeling tools during this century has led to the emergence of new fields of biological research termed systems biology and synthetic biology. The successful exploitation of these developments is evidenced by the creation of increasing number of genetically engineered recombinant cells with superior characteristics (Jantama et al., 2008; Becker et al., 2011) or totally novel functions (Nakamura and Whited, 2003; Yim et al., 2011; Paddon et al., 2013) for diverse sectors such as chemicals and healthcare (Huang et al., 2012; Lee et al., 2012; Sun and Alper, 2014). However, there exists a significant gap in bioprocess performance between studies of the literature and the requirements for an industrially feasible bioprocess for chemical production (Van Dien, 2013). Overall bioprocess performance [productivity (gram/liter/hour), titer (gram/liter) etc.] has to be increased further for successful industrial-scale commercialization to drive the shift from fossil fuel to bioprocess-based chemical production and cost-effective production of novel drugs (Van Dien, 2013). Hence, there is great need for novel approaches addressing these key challenges in chemical and healthcare sectors.

## Potential of Proteome Optimization

With this opinion, we propose that a novel approach of proteome optimization carries a substantial potential for addressing the aforementioned challenges in bioprocess development. That potential arises from the fact that cells express proteins not essential (e.g., flagellar, heat or acid stress proteins) for growth under well-controlled optimal conditions, typically realized in biotechnological processes. This leads to non-efficient use of protein synthesis capacity (translation machinery) and energy for bioprocesses. As translation capacity is believed to be one of the growth-limiting factors, at least in the bacterium *Escherichia coli* (Klumpp et al., 2013), synthesis of non-essential proteins sequesters ribosomes potentially lowering the synthesis capacity of target molecule production. Thus removing the expression burden of non-essential proteins, i.e., creation of lean-proteome strains, could enable to specifically manipulate the allocation of ribosomes for higher synthesis of proteins leading to increased target molecule production. Optimization of the cellular proteome through experimental testing of strains with optimized expression of non-essential proteins and inclusion of protein synthesis capacity constraints in metabolic modeling could open a new avenue for the creation of superior cell factories.

Initial experimental confirmation of the potential of optimization of the layer of protein synthesis capacity for increasing the maximum specific growth rate (μ_max_) of cells comes from two studies of *E. coli* investigating the effects of heterologous protein expression on μ (Scott et al., 2010; Bienick et al., 2014). Both studies show for several heterologous proteins (e.g., LacZ, eGFP) that increasing their expression has a linear negative effect on μ. Their data suggest that for expression of every 1% of heterologous protein per dry cell weight, μ decreases by ~3%. It would be sensible to assume that a similar correlation would exist for the opposite case – decreasing the fraction of non-essential proteins by 1% would lead to an increase in μ by ~3%. Our proposal is also supported by two studies of *Bacillus subtilis* showing that reducing the expression load of proteins non-essential under bioprocess conditions by ~9% fraction from the total proteome through the deletion of the flagellar/motility regulator gene *sigD* leads to a ~30% increase of both μ_max_ and biomass yield (Fischer and Sauer, 2005; Muntel et al., 2014). Further support comes from recent experiments of D’Souza et al. (2014), which show that deletion of single amino acid, vitamin, or nucleobase biosynthesis genes from *E. coli* results in higher μ_max_ compared to the wild-type strain when both strains are grown on medium containing the amino acid, vitamin, or nucleobase that the deletion strain was auxotrophic for. These observations are consistent with earlier chemostat studies with *B. subtilis* (Zamenhof and Eichhorn, 1967) and *E. coli* (Dykhuizen, 1978) where mutants impaired in tryptophan biosynthesis demonstrate significant fitness advantages in the presence of tryptophan relative to prototrophic cells. More importantly, D’Souza et al. (2014) show that deleting genes with higher protein expression cost leads to a greater growth advantage.

The results presented above suggest that proteome resource optimization through decreasing the fraction of non-essential proteins could lead to faster growth and thus also to better bioprocess performance. For instance, target molecule productivity could be increased in growth-coupled production processes by enabling faster growth at the same expression level(s) of target molecule production-related proteins. On the other hand, recombinant protein titers could be significantly elevated by allocating more proteome resources for target protein expression at the expense of lower synthesis of non-essential proteins even at the same μ and/or protein synthesis rate.

## Reduced-Genome Approaches

A conceptually similar approach of creating reduced-genome strains for industrial purposes has been applied in few cases before (Pósfai et al., 2006; Mizoguchi et al., 2008; Unthan et al., 2014; Xue et al., 2014). However, these efforts concentrated on reducing the genome and neglected the effects of gene deletions on the cellular proteome. The approach of deleting large chunks of the genome, instead of specific genes, based on gene function and not on protein abundance was probably responsible for the observed minor positive effects on cellular growth and target molecule production. While the latter studies focused on large-scale genome reduction, experimental technologies enabling more targeted and accurate engineering of strains with reduced load of gene expression have recently emerged. Hence, now the successful execution of the concept of targeted optimization of the layer of protein synthesis capacity is feasible due to the recent rapid progress in proteome-wide absolute quantitative proteomics (Arike et al., 2012; Ahrné et al., 2013; Wiśniewski et al., 2014) and high-throughput genome engineering technologies [e.g., Multiplexed Automated Genome Engineering (MAGE; Wang et al., 2009), trackable multiplex recombineering (TRMR; Warner et al., 2010)]. Thus, the time is ripe to design and create lean-proteome strains possibly leading to superior bioprocess performance.

## Challenges with Proteome Optimization

The main challenge with creating lean-proteome strains is hitting the correct genes/proteins, i.e., genes, which deletion does not lead to detrimental effects. This is a serious concern even in the most studied bacterium *E. coli* since functions for a third of its proteins are still unknown (Keseler et al., 2013) while only ~300 proteins are considered essential for *E. coli* (http://ecoliwiki.net/colipedia/index.php/Essential_genes). It is important to point out that knowing functions/essentiality for more proteins is not the objective *per se* – it is actually more important to know the functions/essentiality of the proteins with the biggest translational burden (abundance × length), as their deletion presumably leads to stronger effects. The good news here is that for many organisms, the proteome mass (a good proxy for length) distribution follows the Pareto principle – ~20% of proteins make up ~80% of the proteome mass (Ghaemmaghami et al., 2003; Maier et al., 2011; Schmidt et al., 2011; Valgepea et al., 2013). Thus, instead of targeting hundreds of genes/genome areas like in the reduced-genome approach described above, one could theoretically greatly increase the key metrics of bioprocess performance (titer, yield, productivity; Van Dien, 2013) by deleting as few as ~10 non-essential genes with the highest translational burden in *E. coli* (in total 7% of proteome; Valgepea et al., 2013) and substituting the “freed” 7% of the total proteome with target molecule-related proteins. Importantly, current mass-spectrometric techniques of absolute proteome quantification (Arike et al., 2012; Ahrné et al., 2013; Wiśniewski et al., 2014) are accurate enough to determine the proteins with the biggest translational burden on the whole-proteome level.

## Strategies of Proteome Optimization for Creating Lean-Proteome Strains

The first and most important step toward creating lean-proteome strains is absolute quantitative proteome analysis of the initial recombinant strain. Accurate characterization of the full proteome is needed for the compilation of lists of non-essential target proteins with the biggest translational burden. We propose two strategies for creating superior lean-proteome strains by targeting proteins with the biggest translational burden, currently specifically for *E. coli*:

1. The first strategy targets proteins with known functions and presumably unnecessary under optimal bioprocess conditions, e.g., pH, temperature, oxygen tension control; defined substrate feed; stirring. These could be proteins involved in stress responses (acid, heat, and osmotic shock), alternative substrate transport and catabolism and cellular movement (flagellar).
2. The second strategy targets proteins with unknown functions with the biggest translational burden. Beneficial for both approaches is the growth screen of all the Keio collection single (Baba et al., 2006) and double deletion strains (personal communication with Prof. Hirotada Mori) that can be used to determine the genes/proteins, which should and should not be targeted.

Another important step is the experimental construction of lean-proteome strains and selection for better production strains. Instead of reducing the proteome one protein at a time, one should target tens of genes with an approach similar to MAGE (Wang et al., 2009), which constantly generates genetic heterogeneity in the pool of mutants allowing the generation of thousands of lean-proteome strains within a few days. The challenge of selecting for better production strains could be tackled by combining several screening methods. First, one could screen for fast growth as reduction of non-essential protein expression should lead to faster growth. Second, high-producing strains could be isolated using fluorescence activated cell sorting (FACS) using a sensor system based on a fluorescent readout corresponding to target molecule levels.

## Potential of Metabolic Modeling

Lastly, one would greatly benefit from an *in silico* metabolic model, which would enable quantitative prediction of the effects of removing non-essential proteins on target molecule production. This should be a model, which incorporates the cellular proteome with the two central features of regulation of μ – cell geometry and cell cycle – and ties the latter to the fluxes of flux balance analysis (FBA)-type models for *in silico* analysis and design of lean-proteome strains. Recently, we have seen serious progress into this direction by the development of a novel single-cell model (Abner et al., 2013), next-generation FBA-type of genome-scale models of metabolism and gene expression (O’Brien et al., 2013; Liu et al., 2014), and a whole-cell model (Karr et al., 2012). Surely, these models will be advanced further and hopefully they will also be able to determine which genes/proteins to delete for creating superior lean-proteome strains.

## Conclusion

Based on the recent rapid advances in high-throughput mutant generation and proteomics technologies together with the emerging novel whole-cell modeling approaches, we conclude that the time is ripe for the metabolic engineering community to directly focus on proteome optimization leading to the creation of lean-proteome strains with superior target molecule production characteristics.

## Conflict of Interest Statement

The authors declare that the research was conducted in the absence of any commercial or financial relationships that could be construed as a potential conflict of interest.
